# Multidetector CT Enterography versus Double-Balloon Enteroscopy: Comparison of the Diagnostic Value for Patients with Suspected Small Bowel Diseases

**DOI:** 10.1155/2016/5172873

**Published:** 2015-12-28

**Authors:** Jingjing Wang, Qiaozhen Guo, Jianping Zhao, Mei Liu, Guangquan Liao, Nianjun Chen, Dean Tian, Xiaoli Wu

**Affiliations:** ^1^Department of Gastroenterology, Tongji Hospital, Tongji Medical College, Huazhong University of Science and Technology, 1095 Jiefang Avenue, Wuhan, Hubei 430030, China; ^2^Hepatic Surgery Center, Tongji Hospital, Tongji Medical College, Huazhong University of Science and Technology, China

## Abstract

*Aim*. To compare the diagnostic value of multidetector CT enterography (MDCTE) and double-balloon enteroscopy (DBE) for patients with suspected small bowel diseases. *Methods*. From January 2009 to January 2014, 190 patients with suspected small bowel diseases were examined with MDCTE and DBE. The characteristics of the patients, detection rates, diagnostic yields, sensitivity, specificity, positive predictive value, and negative predictive value were described and analyzed. *Results*. The overall detection rates of DBE and MDCTE were 92.6% and 55.8%, respectively (*P*<0.05), while the overall diagnostic yields were 83.2% and 33.7%, respectively (*P*<0.05). The sensitivity, specificity, positive predictive value, and negative predictive value of DBE were all higher than those of MDCTE. DBE had a higher diagnostic yield for OGIB (87.3% versus 20.9%, *P*<0.05). The diagnostic yields of DBE were higher than those of MDCTE for inflammatory diseases, angioma/angiodysplasia, and diverticulums, while being not for gastrointestinal tumors/polyps. *Conclusions*. The diagnostic value of DBE for small bowel diseases is better than that of MDCTE as a whole, but if gastrointestinal tumors are suspected, MDCTE is also needed to gain a comprehensive and accurate diagnosis.

## 1. Introduction

Small bowel is the longest viscera in the digestive tract. It is very important for our digestion and absorption. However, due to its special anatomy, the exact examination of small bowel is difficult. With the development of technology, several kinds of examinations have come up and became widely used, among which multidetector CT enterography (MDCTE) and double-balloon enteroscopy (DBE) are important ones.

MDCTE is noninvasive, and it has the advancements of large scanning range, fast collecting, obtaining of multiphase images after a single intravenous injection, and strong image processing ability. Moreover, it allows visualization of the entire small bowel and elimination of artifacts related to respiratory motion with a single breath-hold. With the scanning of multiphase contrast enhancement, MDCTE can acquire images during separate vascular phases, increasing diagnostic potentials [1]. Meanwhile, with the visualization of the entire abdomen, MDCTE can obtain the information of the extraintestinal pathologies. DBE was introduced 13 years ago [2]. Compared with MDCTE and capsule endoscopy, DBE is more visualized and allows repeated observations. It is now the reference standard examination in the small bowel, allowing inspection of the whole small bowel, tissue sampling for histology, and therapeutic endoscopic procedures [3, 4]. However, we found that sometimes the diagnosis of DBE was not accurate and even had no positive findings for “real” patients in clinical work, while MDCTE could draw a diagnosis. What is more, DBE was always compared with other examinations as the goal standard in previous studies. The diagnostic value of DBE itself was evaluated rarely. This paper compared the diagnostic value of MDCTE and DBE by analyzing the data of 190 patients with suspected small bowel diseases.

## 2. Patients and Methods

### 2.1. Patients

This retrospective study was performed at an academic hospital. In the endoscopy center of our institution, we search the searchable database of endoscopies with the phrases “department of gastroenterology” and “double-balloon enteroscopy” and the date from 1st January 2009 to 31st January 2014. 230 patients had been found in total. Seven patients underwent DBE more than one time (1 patient four times and the others two times). Only the first DBE was used for further analysis and 9 repeated patients were excluded. The data (sex, age, indications and reports of DBE and MDCTE, etc.) of 221 patients were collected. All the patients had undergone MDCTE. 31 patients had no clear diagnoses and were excluded. The data of 190 patients were further analyzed. The protocol was approved by the institutional review board at our hospital.

### 2.2. Multidetector CT Enterography

The facility of MDCTE was 64-slice CT750 HD, a product of General Electric Company. The scanning parameters were as follows: 100 KV, 450 mA, pitch 0.984, and slice thickness of 5 mm. Patients would be asked to fast for 12 hours and drink 1500 mL of water before the examination to make the luminal well distensile. The scanning of dual-phase contrast enhancement was carried out 30 s and 60 s after the injection of contrast agent (iopamidol 80 mL, injection speed 3 mL/s), collecting images of arterial phase and portal venous phase. During the procedure, patients would be asked to hold breath to improve the quality of the images.

### 2.3. Double-Balloon Enteroscopy

The DBE was performed with Fujinon EN-450 P5 (Inc., Japan). Like MDCTE, patients should fast for 12 hours before examination. Bowel cleansing carried out by polyethylene glycol electrolytes powder was required. The bowel was considered well cleaned if the stool was watery. Patients were generally anesthetized with fentanyl and propofol during the examination. The starting route was chosen according to the probable location of the lesion. The indication of stopping intubating was the discovery of the lesion, or the realignment was done by two approaches. If the bowel was too narrow to get through, the procedure would be stopped as well.

### 2.4. Protocol

We defined detection rate as the proportion of patients with positive findings by the examination and diagnostic yield (or diagnostic accuracy) as the proportion of patients whose diagnoses by the examination were in accordance with the standard diagnoses. The standard diagnoses were the pathological diagnoses of surgery or biopsy. For the differential diagnosis of Crohn's disease and intestinal tuberculosis, the standard diagnoses were acquired by follow-up (by means of diagnostic treatment). Some standard diagnoses were made by the diagnostic criteria in clinical work (like connective tissue disease, pancreatitis, functional gastrointestinal disorders, etc.). All the data analysis related to the diagnostic yield was done compared with the standard diagnoses. The major adverse events during or after the DBE procedure were defined as perforation, major bleeding, and acute pancreatitis. Minor adverse events were defined as mucosal superficial lacerations and transient intussusception.

### 2.5. Statistical Analysis

The data was analyzed by IBM SPSS Statistics 21 software. Results are expressed as mean ± standard deviation. McNemar's *χ* ^2^-test was used to evaluate the differences between MDCTE and DBE. *P* < 0.05 was considered to be significant.

## 3. Results

190 patients (118 men and 72 women, median age 42.5 years, range from 11 to 82) underwent both MDCTE and DBE. 312 DBE procedures were carried out in total, among which 173 procedures were by oral route and 139 by anal route. The depth of insertion into the small bowel was 108 ± 72 cm (range 10–400 cm) with oral route and 106 ± 87 cm (range 20–300 cm) with anal route. No patients had any adverse events after the procedure. The indications for the examination were summarized in Table 1, showing that the most common indications were abdominal pain and obscure gastrointestinal bleeding (OGIB).

Both the overall detection rate and diagnostic yield of DBE were higher than those of MDCTE (Tables 1 and 2). The statistically significant difference mainly focused on the OGIBs. In patients with abdominal pain, the diagnosing ability of DBE was also better than that of MDCTE, while the detecting ability was not. For patients with other indications, the difference was not statistically significant. The diagnoses were summarized in Table 3, showing that the inflammatory diseases were the most common in small bowel followed by gastrointestinal tumors/polyps. The diagnostic yield of DBE was observably higher than that of MDCTE in inflammatory diseases and angioma/angiodysplasia. DBE diagnosed all the diverticula, while the diagnostic yield of MDCTE was just 12.5%.

DBE had better sensitivity, specificity, positive predictive value, and negative predictive value than MDCTE for suspected small bowel diseases (Table 4). Some points should be mentioned. 10 patients were diagnosed with Crohn's disease (CD) in total. Two CDs were not found by MDCTE while DBE diagnosed all of them. Both MDCTE and DBE diagnosed another 6 patients as CD mistakenly. In patients with tumors, 2 metastatic tumors and 1 pancreatic tumor were all diagnosed by MDCTE, while they were not found by DBE.

## 4. Discussion

The results of the study show the significant difference of the diagnosing ability between MDCTE and DBE. DBE, as a whole, is superior to MDCTE in detecting lesions and diagnostic accuracy. Though the *P* value of detection rate could not be calculated for OGIBs, it can be seen that DBE did an obviously better job for OGIBs than patients with other indications. Thus, the much higher number of OGIB patients may be responsible for the higher overall detection rate and diagnostic yield of DBE. DBE may have no obvious advantage compared with MDCTE in patients with other indications, but we could not make an exact conclusion considering the small sample number. Meanwhile, there are few literatures comparing the diagnostic value of MDCTE and DBE in patients with abdominal pain, diarrhea, and so on. It still needs further study.

A few studies had evaluated the diagnostic value of DBE and MDCTE for OGIB. The reported diagnostic yield of DBE for OGIB ranges from 60% to 81% [5], while that of MDCTE varies from 24.6% to 47.6% [6–8]. A study from Yen et al. [9] also showed that the diagnostic yield of DBE is higher than that of MDCTE with statistical significance (93.5% versus 45.2%, *P* < 0.05) comparable to the results in our study. Interestingly, the diagnostic yield of MDCTE for OGIB depends on the etiology. Previous studies have shown that MDCTE has a high diagnostic yield of tumor versus nontumor etiology of OGIB, while it is not so sensitive in diagnosing flat mucosal lesions, such as superficial ulcers or erosions and vascular abnormalities [9–11]. In this study, the results are comparable with the diagnostic yield of MDCTE for inflammatory diseases being significantly lower than that of DBE (25.0% versus 90.2%, *P* < 0.05). More than one study has verified the diagnostic value of MDCTE for OGIB, for it has multiple phases and can detect gastrointestinal tract bleeding and identify the source [11, 12]. However, due to its insufficiency in detecting superficial ulcers or erosions and vascular abnormalities, we cannot definitely exclude possible small bowel diseases with negative MDCTE findings [7, 10]. The especially low negative predictive value of MDCTE in our study also supported the point. It would be the best to combine MDCTE with DBE. The study from Yen et al. has also shown that positive MDCTE results would make it easier for DBE to choose the correct insertion route than negative MDCTE results (100% versus 52.9%, *P* = 0.003). It is suggested that MDCTE can be conducted prior to DBE, helping the endoscopy operator to identify the patients who are proper to undergo DBE and to choose the most efficient route of DBE examination [9].

In the study, inflammatory disease was the most common disease, followed by gastrointestinal tumors/polyps and angioma/angiodysplasia. Several studies have reported that these three diseases constitute the top three etiologies of OGIB [13, 14], explaining that they are the three most common diseases in this study, with the OGIB patients being the most included ones.

Crohn's disease is less common in clinical work but usually with difficulty to diagnose. In our study, the number of CDs was so small that we could not calculate some statistical indicators to evaluate the diagnostic value of the two examinations. DBE diagnosed all the CDs. It may be sensitive in finding and diagnosing CD, but also easy to make mistake. Previous study showed that MDCTE can demonstrate changes of active CD, such as mucosal enhancement, wall thickening, the bowel wall edema, and associated mesenteric adverse events [15]. A meta-analysis reported that the diagnostic yield of MDCTE is 21% in patients with suspected CD and 39% in established CD patients [16]. Nevertheless, the diagnosis of small bowel CD is observer and modality dependent on the basis of the study of a substantial number of patients [17]. So it is suggested that the images should be read by the radiologists with experience. DBE has been confirmed to be useful for the diagnosis in suspected or known small bowel CD in early study [18]. The diagnostic yield was 30% in suspected CD and approximately 60% in established CD patients, with a higher yield if the location of the lesion was verified by other investigations previously [19, 20]. The yield is not so satisfactory. It may be the reason that in many conditions the differential diagnosis of CD with other diseases is difficult, for the lesions are not specific for CD [21]. Thus, it requires much of the operator to diagnose CD, and, of course, biopsies will be more relied on. Being a noninvasive diagnostic procedure, one study suggested MR enteroclysis (MRE) to be the prior choice in patients with suspected small bowel CD, followed by capsule endoscopy (CE) and enteroscopy [22]. Nevertheless, the results of a comparative trial showed that CE is superior to MRE in detecting small bowel lesions in CD (83.6% versus 45.5%, *P* < 0.05), especially in proximal and mid-small bowel [4]. In the Digestive Disease Week (DDW) highlights in 2014, MRE and CE are considered to be complementary, while DBE, as the gold standard, should be used to validate CE findings [4].

MDCTE and DBE have their own advantages in diagnosing tumors and polyps. MDCTE is considered to be accurate for detecting small bowel neoplasms, showing the form and the location of the tumor clearly as well as the relationship between the tumor and surrounding tissues [23]. What is more, whether there are metastases and where the metastases are, for example, mesenteric lymph and liver, can be revealed, which have clinical significance for tumor grading and treatment selection [17]. In the present study, 2 metastatic tumors and 1 extraintestinal tumor were all diagnosed by MDCTE, while they were not found by DBE. The main reason may be that DBE cannot obtain the information of extraintestinal tissues. However, the characteristics of DBE make it have both diagnostic and therapeutic potentialities. Meanwhile, the positional mark made by DBE would be of great help for surgeons to locate the lesions [24]. One study has reported that the detection rate and the diagnostic yield of DBE for gastrointestinal mesenchymal tumors were 92.2% and 88.3%, respectively, while the other study reported that the positive detection rate of DBE for small bowel tumors was significantly higher than that of CT scan (85.9% versus 72.9%) [24, 25]. In our study, the diagnostic yields of MDCTE and DBE for gastrointestinal tumors/polyps are lower and not significantly different. The results may be attributed to the small number. Previous studies have suggested DBE to be the gold standard for the diagnosis of small bowel tumors [25, 26]. However, considering the deficiency that DBE cannot give a description of extraintestinal and peritoneal tissues, it may be better to combine MDCTE and DBE for patients with suspected small bowel tumors.

Angioma or angiodysplasia is one of the most common etiologies for OGIB, and the results showed that DBE had obvious advantages over MDCTE in its diagnosing. With the direct visualization of the small bowel, DBE can discover the vascular abnormality easily, while, for MDCTE, it usually presents as enhancing nodule [17], which may be mixed with other lesions. It is difficult for MDCTE to reveal the image of diverticula directly, mostly diverticula shown as inflammatory lesions. Thus, MDCTE is not so good at diagnosing diverticula.

The sensitivity, specificity, positive predictive value, and negative predictive value of DBE were all higher than those of MDCTE, implying that DBE was better at picking out the patients who indeed had small bowel diseases and at detecting the patients who had no organic diseases. It was more credible for DBE than MDCTE to diagnose a patient with no organic disease if the examination had no positive findings. However, only 13 patients, who were diagnosed with functional gastrointestinal disorders, had no organic diseases in total. The number was so small that the results of specificity and negative predictive value may not be general.

Though DBE does a good job in diagnosing small bowel diseases, it is an invasive procedure after all. It costs more time and labor and has a considerable risk of adverse events such as pancreatitis and perforation [11]. Notably, no one had been reported to have any adverse events in our study, suggesting that adverse events can be avoided considerably by an experienced endoscopy operator. MDCTE is noninvasive, but it has radiation exposure. Meanwhile, the condition of bowel distention, gastrointestinal dysmotility, and bowel obstruction may be all the limiting factors of the application of MDCTE [27].

There are some limitations of this study. First, the study is a single-center study, so it is not so generalized. In addition, though the total number of the patients is considerable, the number is small referring to the specific disease, which is not good for the data analysis. Furthermore, as a retrospective study, the examinations were carried out by several different doctors. The differences of the diagnostic ability between the doctors may result in the error of the data analysis.

## 5. Conclusion

In conclusion, DBE is superior to MDCTE in diagnosing small bowel diseases and detecting patients who have no organic gastrointestinal diseases. OGIB is the most common indication for patients to undergo small bowel inspection. DBE has a high diagnostic yield in patients with OGIB, while MDCTE is sensitive in tumor etiology of OGIB and can guide the proper route of DBE. DBE is useful in diagnosing CD, and the difficult differential diagnoses are the main obstructions for a higher diagnostic yield. DBE is the gold standard for the diagnosis of small bowel tumors, while MDCTE is also necessary to obtain the information of the extraintestinal and peritoneal tissues.

## Figures and Tables

**Table 1:** Detection rate in patients with different indications.

Indications	*N* (%)	Detection rate		
		MDCTE	DBE	*P* value
Abdominal pain	53 (27.9%)	37 (69.8%)	45 (84.9%)	0.057

Obscure gastrointestinal bleeding	110 (57.9%)	52 (47.3%)	110 (100%)	—^1^

Chronic diarrhea	7 (3.7%)	17 (63.0%)	21 (77.8%)	0.344
Suspected inflammatory bowel disease	4 (2.1%)			
Suspected gastrointestinal tumors/polyps	5 (2.6%)			
Intestinal obstruction	3 (1.6%)			
Ascites	3 (1.6%)			
Vomit	2 (1.1%)			
Malnutrition	1 (0.5%)			
Abnormal defecation	1 (0.5%)			
Abdominal mass	1 (0.5%)			

Total	190 (100.0%)	106 (55.8%)	176 (92.6%)	<0.05

^1^As the detection rate of DBE for OGIBs was 100% and was considered to be constant quantity in SPSS, *χ* ^2^-test was not applicable.

**Table 2:** Diagnostic yield in patients with different indications.

Indications	MDCTE	DBE	*P* value
Abdominal pain (*n* = 53)	26 (49.1%)	41 (77.4%)	<0.05
Obscure gastrointestinal bleeding (*n* = 110)	23 (20.9%)	96 (87.3%)	<0.05
Others^1^ (*n* = 27)	15 (55.6%)	21 (77.8%)	0.146

Total (*n* = 190)	64 (33.7%)	158 (83.2%)	<0.05

^1^Others include the patients with the following indications: chronic diarrhea (*n* = 7), suspected inflammatory bowel disease (*n* = 4), suspected gastrointestinal tumors/polyps (*n* = 5), intestinal obstruction (*n* = 3), ascites (*n* = 3), malnutrition (*n* = 1), vomit (*n* = 2), abnormal defecation (*n* = 1), and abdominal mass (*n* = 1).

**Table 3:** Diagnostic yield in patients with different diseases.

Diagnoses	*N* (%)	Diagnostic yield		
		MDCTE	DBE	*P* value
Inflammatory diseases^1^	92 (48.4%)	23 (25.0%)	83 (90.2%)	<0.05
Gastrointestinal tumors/polyps^2^	41 (21.6%)	23 (56.1%)	31 (75.6%)	0.096
Angioma/angiodysplasia	19 (10.0%)	5 (26.3%)	15 (78.9%)	<0.05
Diverticulum^3^	16 (8.4%)	2 (12.5%)	16 (100.0%)	—^5^
Others^4^	22 (11.6%)	11 (50.0%)	13 (59.1%)	0.754

Total	190 (100.0%)	64 (33.7%)	158 (83.2%)	<0.05

^1^Inflammatory diseases include ulcers/erosions (*n* = 78), Crohn's disease (*n* = 10), intestinal tuberculosis (*n* = 2), ulcerative colitis (*n* = 1), and inflammatory bowel disease (*n* = 1).

^2^Gastrointestinal tumors/polyps include neoplasm (*n* = 3), gastrointestinal stromal tumors (*n* = 14), lymphoma (*n* = 3), metastatic tumors (*n* = 2), pancreatic tumor (*n* = 1), gastrointestinal carcinoma (*n* = 7), leiomyoma (*n* = 1), multiple prominences (*n* = 1), polyps (*n* = 6), Peutz-Jeghers syndrome (*n* = 2), and familial polyposis (*n* = 1).

^3^Four of the sixteen patients were Meckel's diverticulum.

^4^Others include incomplete intestinal obstruction (*n* = 2), intestinal bleeding (*n* = 1), ancylostomiasis (*n* = 1), connective tissue disease (*n* = 1), anaphylactoid purpura (*n* = 1), ileal duplication (*n* = 1), pancreatitis (*n* = 2), and functional gastrointestinal disorders (*n* = 13).

^5^As the diagnostic yield of DBE for diverticulum was 100% and was considered to be constant quantity in SPSS, *χ* ^2^-test was not applicable.

**Table 4:** 

	MDCTE	DBE
Sensitivity	57.1%	97.7%
Specificity	61.5%	76.9%
Positive predictive value	95.3%	98.3%
Negative predictive value	9.5%	71.4%
